# Xenoreactive antibody response following pulmonary valve replacement using porcine bioprosthesis in the young

**DOI:** 10.1186/1749-8090-8-S1-O138

**Published:** 2013-09-11

**Authors:** MS Kim, YJ Kim, HK Lim, CS Park

**Affiliations:** 1 Department of Thoracic and Cardiovascular Surgery, Seoul National University Hospital, Seoul, Korea; 2 Department of Thoracic and Cardiovascular Surgery, Asan Medical Center, Seoul, Korea

## Background

Xenoreactive antibody reaction is known to initiate the immune-mediated valve destruction. To investigate the immune effect, serum anti-α-Gal antibody response following the pulmonary bioprosthesis implantation, including clinical factors, immunoglobulin types and patterns that might influence the anti-α-Gal immune response in children and young adults were studied.

## Methods

Between January 2008 and February 2011, 40 patients underwent pulmonary valve replacement using a porcine bioprosthesis at an age younger than 30 years. There were 27 males (67.5%), and the median age at operation was 14 years (1.1–27.3 years). Serum was obtained from each patient prior to the operation, 1 day after the operation, at discharge, and at the first and second outpatient clinic visits. These samples were analyzed with an enzyme-linked immunosorbent assay.

## Results

Regardless of the isotype, anti-α-Gal antibody activity was increased at discharge and at the first outpatient visit. Although anti-α-Gal IgG antibody activity remained increased by the second outpatient visit, anti-α-Gal IgM antibody activity did not. Anti-α-Gal IgG antibody activity was higher at discharge among patients younger than 15 years. Anti-α-Gal IgG antibody activity were more prominent at the second outpatient visit in non-blood group B patients (A, O).

## Conclusions

The implantation of a porcine bioprosthesis elicits an increased formation of anti-α-Gal antibodies, with different patterns of IgM and IgG isotypes in children and young adults. Patient’s age and ABO blood group may influence the patterns of anti-α-Gal immune response after pulmonary valve replacement.

The early postoperative xenoreactive immune response could be considered to influence the initial process of degenerative valve failure.

